# Reporting Completeness of Usual Care Comparator Groups in Exercise‐Based Trials for Knee Osteoarthritis: A Meta‐Research Systematic Review

**DOI:** 10.1002/msc.70248

**Published:** 2026-06-28

**Authors:** José Ribeiro da Silva Neto, Lucas Henrique Caldas, Paula Gabrielly Oliveira Demes, Natalia Camargo Rodrigues Iosimuta, Ana Carolina Pereira Nunes Pinto, Areolino Pena Matos

**Affiliations:** ^1^ Faculty of Physical Therapy Federal University of Amapá Macapá Amapá Brazil; ^2^ Postgraduate Program in Health Sciences Federal University of Amapá Macapá Amapá Brazil; ^3^ Institut de Recerca Sant Pau (IR SANT PAU), Iberoamerican Cochrane Centre Barcelona Spain; ^4^ Cochrane Brazil Centre for Evidence‐Based Health Studies and Health Technology Assessment São Paulo São Paulo Brazil; ^5^ Postgraduate Program in Human Movement Sciences Federal University of Pará Belém Pará Brazil

**Keywords:** exercise therapy, health care quality, intervention reporting, knee osteoarthritis, randomized controlled trials as topic

## Abstract

**Background:**

Exercise‐based randomized controlled trials are essential for guiding the management of knee osteoarthritis, but their interpretability depends on transparent reporting of both intervention and comparator groups. The term usual care (UC) is frequently used as a comparator, yet its reporting quality remains unclear.

**Objective:**

To evaluate the reporting completeness of UC comparator groups in randomized controlled trials (RCTs) of exercise‐based interventions for knee osteoarthritis.

**Methods:**

This systematic review included RCTs of exercise‐based interventions for adults with knee osteoarthritis that used UC as a comparator. Searches were conducted in MEDLINE, EMBASE, CENTRAL, PEDro, CINAHL, and SPORTDiscus up to December 2024. Reporting completeness was assessed using the Template for Intervention Description and Replication (TIDieR) checklist and analysed using continuous and dichotomised approaches.

**Results:**

Sixty‐seven RCTs were included. Reporting completeness of UC interventions was consistently low. Median TIDieR scores (range 0–12) were higher for exercise interventions (4.0; IQR 3–5) than for UC comparators (1.0; IQR 1–2). Continuous analyses detected significant differences between groups, whereas dichotomised analyses did not. Only 22.3% of exercise‐based interventions achieved high reporting completeness. No improvement was observed after publication of the TIDieR checklist, and no meaningful association was found between methodological quality and reporting completeness.

**Conclusions:**

Reporting of UC comparator groups in exercise‐based RCTs for knee osteoarthritis is poor and has not improved over time. Although exercise interventions are better reported, overall completeness remains low, highlighting the need for improved reporting practices to support clearer interpretation of clinical trial evidence.

## 1 Introduction

Osteoarthritis (OA) is the most prevalent chronic degenerative joint disease worldwide and represents a major cause of pain, functional limitation and disability among older adults (Hunter and Bierma‐Zeinstra 2019; Li et al. 2024). Knee OA is especially burdensome, affecting an estimated 300 million individuals globally and substantially contributing to years of living with disability (Cross et al. 2014; Prieto‐Alhambra et al. 2014). As population ageing and obesity rates continue to rise, the global impact of knee OA is expected to increase further, reinforcing the need for effective strategies.

Exercise therapy, including resistance, aerobic, aquatic, and mind‐body approaches, has been shown to be effective in reducing pain and improving physical function in individuals with knee OA (Fransen 2015; Mo et al. 2023; Raposo et al. 2021). Accordingly, randomized controlled trials (RCTs) are central to evaluating the effectiveness of exercise‐based interventions and informing clinical guidelines. However, the interpretability and applicability of these trials depend not only on methodological rigour but also on the transparent and complete reporting of all study arms (Arienti et al. 2022; Goh et al. 2019).

In exercise‐based RCTs, comparator groups are frequently labelled as ‘*usual care*’ (UC). Although UC is intended to reflect routine clinical practice, its definition varies substantially across studies and healthcare contexts (Arienti et al. 2022; Goh et al. 2019; Paci et al. 2022; Smelt and Assendelft 2010). Previous studies have shown that UC may include a wide range of components, including education, counselling, pharmacological management, physical therapy, lifestyle advice, or even no intervention (Abbott et al. 2013; Arienti et al. 2022; Biesecker et al. 2021; Smelt and Assendelft 2010; Thompson and Schoenfeld 2007). This heterogeneity reflects real‐world clinical variability but also introduces ambiguity in the interpretation of trial findings, limiting reproducibility and complicating evidence synthesis.

Emerging evidence from meta‐research and methodological studies indicates that comparator group characteristics can influence effect size estimates and potentially bias conclusions regarding intervention effectiveness. Studies conducted in different clinical areas, including musculoskeletal conditions and rehabilitation, have demonstrated that UC comparator groups are often poorly defined and inconsistently reported (Yu et al. 2018; Paci et al. 2022). In the context of knee OA, prior systematic reviews have highlighted that variability in comparator interventions contributes to heterogeneity in treatment effects, further underscoring the importance of clearly describing all trial arms.

Despite these concerns, the reporting of UC comparator groups in exercise‐based RCTs for knee OA has not been systematically evaluated. Although reporting guidelines such as the template for Intervention Description and Replication (TIDieR) checklist (Hoffmann et al. 2014) aim to improve the completeness and transparency of intervention descriptions, their impact on the reporting comparator groups, particularly for unstructured or pragmatically defined interventions, remains unclear.

Previous research has further highlighted these limitations. UC interventions are highly variable and often poorly described, particularly in musculoskeletal and primary care settings (e.g., Marriott 2024; K. M. Turner et al. 2024). In addition, evidence from knee osteoarthritis trials suggests that treatment effect estimates may vary substantially depending on the comparator used (Adu et al. 2025; Pedersen et al. 2023). However, there is a lack of systematic evaluation focused on the completeness of reporting of UC comparator groups using standardised frameworks such as TIDieR. Furthermore, it remains unclear how reporting completeness differs between UC and active interventions, and whether it has improved over time or is associated with methodological quality.

Adopting a meta‐research perspective, this systematic review aims to evaluate the reporting completeness of UC comparator groups in randomized controlled trials of exercise‐based interventions for knee osteoarthritis using the TIDieR checklist. Additionally, it compares reporting between UC and exercise interventions, explores temporal trends in reporting quality, and examines associations with methodological quality.

## 2 Methods

### 2.1 Registration

The study protocol was prospectively registered on the Open Science Framework (OSF) under the identifier (10.17605/OSF.IO/R7MFC). A detailed version of the protocol was also published in *Fisioterapia Brasil* (https://doi.org/10.62827/fb.v25i1.j161).

### 2.2 Study Design

A meta‐research systematic review was conducted to evaluate the reporting completeness of usual care (UC) comparator groups in randomized controlled trials of exercise‐based interventions for knee OA. The study was designed and reported in accordance with the Preferred Reporting Items for Systematic Reviews and Meta‐Analyses (PRISMA) guidelines and followed the recommendations from the Cochrane Handbook for Systematic Reviews of Interventions.

### 2.3 Eligibility Criteria

Studies were included if they were randomized controlled trials evaluating exercise‐based interventions for treating knee OA, in individuals with knee osteoarthritis diagnosed based on clinical and/or radiographic criteria. Exercise‐based interventions were defined as structured, planned, and repetitive physical activities aimed at improving pain, physical function, or related outcomes (resistance, aerobic, aquatic, or mind–body exercise).

Eligible studies were required to include a comparator group described as UC or synonymous terms (e.g., standard care, routine care, conventional treatment, education, follow‐up, or treatment not described) as mentioned in previous studies (Abbott et al. 2013; Arienti et al. 2022; Biesecker et al. 2021; Smelt and Assendelft 2010; Thompson and Schoenfeld 2007).

Comparator groups were included based on the terminology used by the original study authors (e.g., ‘usual care,’ ‘standard care’), regardless of their specific content, in order to reflect the heterogeneity and conceptual ambiguity of UC in clinical trials. This approach was chosen to reflect real‐world practice.

Studies were excluded if they were non‐randomized, partially randomized, or not available in full text.

### 2.4 Search Strategy

A comprehensive search strategy was conducted in the following electronic databases: MEDLINE (via PubMed), Embase (via Elsevier), Cochrane Library (via CENTRAL), PEDro, SportDiscus, and CINAHL.

The search was conducted from database inception to 31 December 2024 with no restrictions on language or publication date. The inclusion of studies published in 2024 reflects database indexing timing and an updated search conducted prior to the final analysis.

Search terms combined controlled vocabulary and keywords related to knee osteoarthritis, exercise‐based interventions, and randomized controlled trials. Although the primary focus of this review was on UC comparator groups, terms related to exercise were included to ensure appropriate identification of eligible trials.

The full search strategy for each database is provided in Supporting Information S1.

### 2.5 Study Selection

All records were imported into the Rayyan platform for screening (https://www.rayyan.ai). Two independent reviewers performed the study selection in a three‐step process: (1) identification of records, (2) screening of titles and abstracts, and (3) full‐text assessment. Disagreements were resolved through discussion with a third reviewer.

The search strategy was designed to identify randomized clinical trials that included the UC comparator in knee OA interventions. To contextualise the studies, terms related to exercise‐based interventions such as exercise, therapeutic exercise, and physical activity were incorporated, recognising that these concepts are not strictly equivalent. Study eligibility was defined by the presence of a comparator group described as UC, irrespective of the terminology used to define the intervention group. As this review evaluated the quality of reporting of the UC comparator groups, the analysis centred on this group, independent of how the intervention group was labelled.

### 2.6 Data Extraction

Data were independently extracted by two reviewers, including study characteristics (authors, year, country) authorship, sample characteristics, description of exercise interventions, description of UC comparator groups, TIDieR scores for both comparator and exercise groups and PEDro scores. For studies not indexed in the PEDro database, methodological quality was independently assessed by the reviewers. Disagreements were resolved by consensus.

### 2.7 Assessment of Methodological Quality

The methodological quality was assessed using the PEDro scale, which consists of 11 items, 10 of which are scored. Based on the total score, studies were classified as poor (< 4), fair (4–5), good (6–8), or excellent (9–10). The risk of bias was categorised as high (≤ 3), moderate (4–5), or low (≥ 6). When available, PEDro scores were obtained directly from its database (de Morton 2009); otherwise, they were assessed manually by trained reviewers.

### 2.8 Assessment of Reporting Completeness

The reporting completeness of both exercise interventions and UC comparator groups was assessed using the TIDieR checklist, which includes 12 items covering key aspects of intervention description. Each item was scored as 1 = adequately reported or 0 = partially reported or not reported. Total scores range from 0 to 12, with higher scores indicating more complete reporting. TIDieR scores were converted into percentages using the formula: (Score × 100)/12.

Reporting completeness was additionally categorised as: high quality (≥ 50%) or low quality (< 50%), This threshold was defined a priori by the authors based on previous meta‐research (Barros et al. 2022) and is not intended to represent an established universal standard.

The year 2014 was selected as a reference point based on the publication of the TIDieR checklist. Additionally, temporal trends were explored using the year of publication as a continuous variable to capture gradual changes over time.

Although the TIDieR checklist was originally developed for structured interventions, it was applied in this study to both intervention and comparator groups to allow standardised assessment of reporting completeness. All reviewers underwent prior training in the use of the assessment tools and a pilot phase was conducted to ensure consistency in scoring procedures.

### 2.9 Statistical Analysis

Data distribution was initially assessed using the Shapiro–Wilk test. As most variables demonstrated non‐normal distributions, continuous data are presented as medians and interquartile ranges (IQR). Between‐group comparisons (exercise vs. usual care) were conducted using the Mann–Whitney *U* test.

Categorical variables were reported using absolute and relative frequencies and compared using chi‐square tests when appropriate. Continuous TIDieR scores were considered the primary analytical approach, as they preserve the full variability and provide greater sensitivity to differences in reporting completeness. Dichotomised analysis (≥ 50% vs. < 50%) were conducted as secondary, exploratory analysis to enhance the interpretability. However, this approach may reduce the data granularity and statistical power.

Inter‐rater agreement was assessed using the Kappa coefficient, interpreted as: almost perfect (0.81–1.00), substantial (0.61–0.80), moderate (0.41–0.60), fair (0.21–0.40), slight (0.00–0.20), or poor (< 0.00) (Landis and Koch 1977).

The consistency between TIDieR and PEDro scores was examined using the intraclass correlation coefficient (ICC), interpreted as: poor (< 0.50), moderate (0.50–0.75), good (0.75–0.90), or excellent (> 0.90) (Koo and Li 2016). All analyses were performed using JAMOVI software, with the significance level set at *p* < 0.05. Reporting completeness (TIDieR), methodological quality (PEDro), and risk of bias were analysed as distinct constructs.

## 3 Results

**FIGURE 1:**
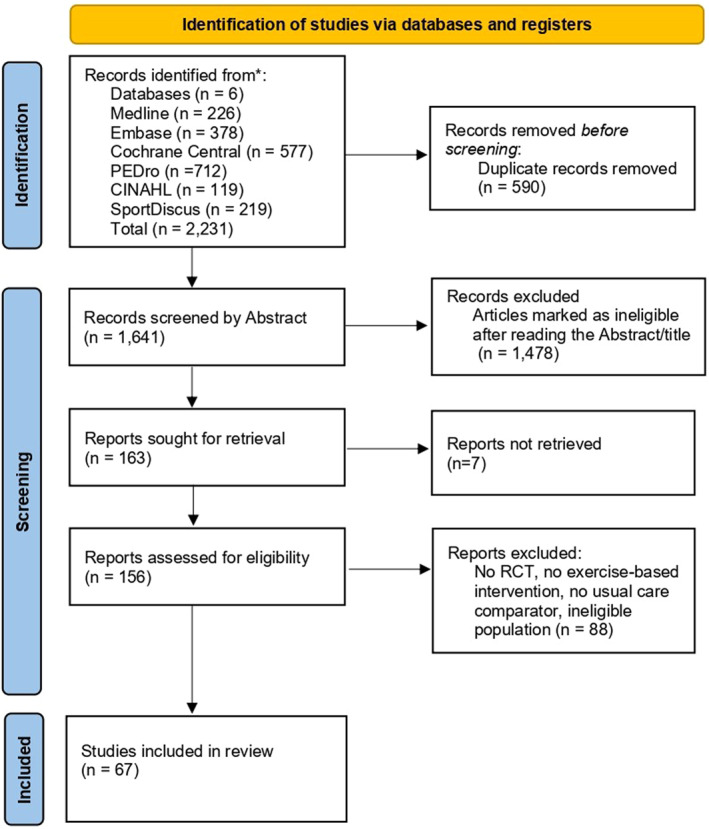
PRISMA flow diagram of study selection.

The database search retrieved 2231 records. After removal of duplicates, titles and abstracts were screened, followed by full‐text assessment of potentially eligible studies. A total of 67 randomized controlled trials (RCTs) met the inclusion criteria and were included in this systematic review. The study selection process, including detailed reasons for exclusion at the full stage, is presented in a PRISMA flow diagram (Figure 1).

### 3.1 Characteristics of Included Studies

The included RCTs were published between 1992 and 2024 and evaluated a wide range of exercise‐based interventions for individuals with knee osteoarthritis. Across studies, UC comparator groups were described using heterogeneous terminology, including ‘usual care,’ ‘standard care,’ ‘routine care’ and related terms.

Among the descriptions of the UC comparator group, only 31.3% (21/67) reported an explicit operational definition. In the remaining trials (68.7%), UC was described using generic, non‐specific terms or mentioned without any descriptions of its components. The general characteristics of the included studies are presented in Table 1.

**TABLE 1:** Overall characteristics and reporting completeness of usual care (UC) comparator groups in the included randomized controlled trials.

Characteristic	Description
Number of trials	67 randomized controlled trials
Exercise intervention type	Strengthening, aerobic, mixed exercise
Comparator labels used	Usual care, standard care, routine care
UC description used	100% (67/67)
UC explicitly defined (operational description)	31.3% (21/67)
UC poorly defined (generic description)	68.7% (46/67)
Median TIDieR score UC groups	1 (IQR 1–2); (possible range 0–12)
UC classified as high reporting completeness (≥ 50% TIDieR items)	0% (0/67)
Follow‐up duration	6 weeks to 24 months

### 3.2 Reporting Completeness of Usual Care Comparator Groups

The overall reporting completeness of the UC comparator groups was low. TIDieR observed scores ranged from 1 to 5 with a median of 1.0 (IQR 1–2). No study achieved moderate or high reporting completeness (≥ 6 items).

Across individual TIDieR items, basic elements such as the intervention name (Item 1) were consistently reported, whereas key components, such as materials, procedures, and delivery details were infrequently described. Items related to intervention fidelity (Items 11 and 12) were rarely reported.

Detailed descriptions of the UC comparator groups and item‐level TIDieR scores for each included study are provided in Table S1.

### 3.3 Comparison Between Exercise and Usual Care Reporting

**FIGURE 2:**
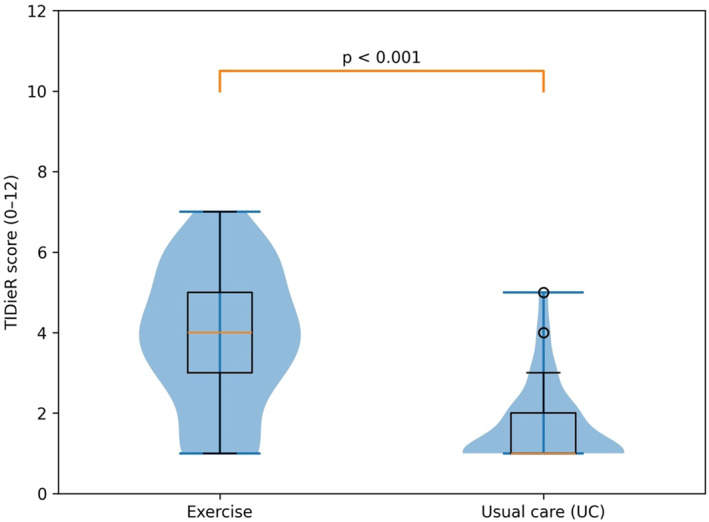
Reporting completeness of interventions according to TIDieR checklist. Violin plots with overlaid boxplots showing the distribution of TIDieR scores for exercise‐based interventions and usual care (UC) comparator groups. The *p*‐value refers to the Mann–Whitney *U* test comparing groups.

Exercise‐based interventions demonstrated significantly higher reporting completeness than the UC comparator groups. The median TIDieR score was 4.0 (IQR 3–5) for exercise interventions and 1.0 (IQR 1–2) for UC comparators (*p* < 0.001) as shown in Figure 2. Despite this difference, overall reporting quality remained limited in both groups. Only 22.3% of exercise interventions achieved high reporting completeness (≥ 50%).

When reporting completeness was dichotomised into high and low categories, no statistically significant difference was observed between groups (*p* = 0.061), suggesting that categorical classification may underestimate differences captured by continuous scores.

### 3.4 Item‐Level Reporting Patterns

Both exercise and UC groups showed similar patterns in the most and least frequently reported TIDieR items. The most commonly reported items included: Item 1 (Brief name): 100% in both groups; Item 3 (Materials): 49.2% in exercise versus 17.9% in UC; Item 4 (Procedures): 47.7% in exercise versus 13.4% in UC. The least frequently reported items were: Item 11 (How well planned): 3.0% in exercise versus 1.5% in UC; Item 12 (How well actual): 1.5% in both groups. These findings indicate that critical aspects related to intervention fidelity are consistently underreported across both intervention and comparator groups.

### 3.5 Temporal Trends in Reporting Quality

**FIGURE 3:**
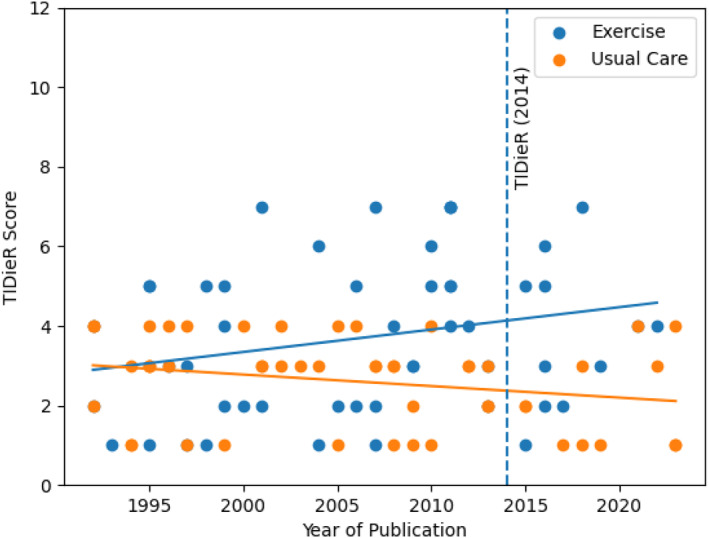
Temporal trends in reporting completeness according to the TIDieR checklist. Scatter plot showing the relationship between the year of publication and TIDieR scores for exercise‐based interventions and usual care (UC). Each point represents an individual study. Regression lines indicate trends over time.

No clear temporal improvement in reporting completeness was observed for either exercise‐based interventions or UC comparator groups. For exercise interventions, the median was 4.0 (IQR 3–5) before 2014 and 4.0 (IQR 2.75–5.25) after 2014 (*p* = 0.708). Similarly, the UC comparator groups showed identical median scores of 1.0 (IQR 1–2) before and after 2014 (*p* = 0.669) (Figure 3).

### 3.6 Methodological Quality

Based on the PEDro scale, most studies demonstrated moderate to high methodological quality. Specifically, 26.8% were classified as fair, 71.6% as good and 1.5% as excellent. Regarding risk of bias, it was observed that 26.8% of the studies were classified as moderate risk and 73.1% as low risk.

### 3.7 Reliability and Association Analyses

Inter‐rater reliability was substantial for both PEDro and TIDieR assessments. The intraclass correlation coefficient (ICC) for PEDro scores was 0.68 (95% CI: 0.57–0.76), and for TIDieR scores was 0.67 (95% CI: 0.43–0.70). The association between methodological quality (PEDro) and reporting completeness (TIDieR) was weak and non‐significant. The agreement between PEDro and TIDieR exercise scores was poor (ICC = 0.29; 95% CI: −0.14 to 0.56; *p* = 0.08), and between PEDro and TIDieR UC scores was also poor (ICC = −0.48; 95% CI: −0.70 to 0.35; *p* = 0.57). These findings indicate that methodological rigour and reporting completeness represent distinct dimensions of trial quality.

## 4 Discussion

Knee osteoarthritis is one of the most common musculoskeletal conditions worldwide, causing pain and disability. Exercise‐based interventions are consistently recommended as first line treatment in clinical guidelines, supported by numerous randomized clinical trials (RCTs). However, interpreting these trials depends not only on the quality of intervention but also on the adequacy and transparency of the comparator groups, particularly those described as UC.

In this systematic review, we evaluated the completeness of reporting of UC comparator groups in RCTs of exercise‐based interventions for knee OA using the TIDieR checklist, and examined differences between groups, temporal trends, and associations with methodological quality. We found that a median TIDieR score of 1.0 (IQR 1–2), with no study achieving moderate or high completeness. Although exercise interventions demonstrated relatively higher scores, their reporting remained suboptimal, indicating that incomplete reporting is not restricted to comparator groups. Previous studies have similarly reported suboptimal reporting of exercise interventions in knee osteoarthritis trials (Bartholdy et al. 2019), suggesting a broader issue in the field. Accordingly, our findings on UC should be interpreted within this context. Importantly, no improvement in reporting was observed over time, even after the publication of the TIDieR checklist (Hoffmann et al. 2014).

These findings have important methodological implications. Comparator groups play a critical role in determining the magnitude of treatment effects, and inadequate reporting limits the ability to interpret whether observed differences are from the intervention or to variability in the comparator (Coates 2010; Knoop et al. 2020; Mete and Sari 2022; Rewald et al. 2020; Smelt and Assendelft 2010). Although our findings support the plausibility of this mechanism, we did not directly examine the relationship between reporting completeness of UC and treatment effect estimates. Evaluating whether poorly reported or more heterogeneous comparator groups are associated with larger or more variable effect sizes represents an important direction for future research and could further clarify the methodological implications of inadequate comparator reporting.

This lack of transparency may compromise internal validity, limit reproducibility, and introduce bias in evidence synthesis, particularly in meta‐analyses where comparator heterogeneity is often insufficiently addressed (Dechartres et al. 2013; Hrobjartsson et al. 2012). Previous research has shown that low‐quality reporting of comparator groups can bias effect size estimates and mislead interpretation (Dawson et al. 2009; de Bruin 2010; Freedland et al. 2011).

A key finding of this review was the substantial heterogeneity in how UC was defined and implemented. Terms such as ‘*usual care*’, ‘*standard care*’ or ‘*routine care*’, were frequently used; however, these labels encompassed highly variable components, including medical consultations, physical therapy, education, pharmacological treatment, or even no intervention (Brännström and Boman 2014; Hunter et al. 2023; Knoop et al. 2020; Rêgo et al. 2023; Temel 2010; K. M. Turner et al. 2024). In many trials, UC was not explicitly defined, rendering the term ‘usual’ ambiguous and potentially misleading. Similar challenges have been reported in other clinical fields, suggesting that this is a persistent methodological issue rather than one specific to knee osteoarthritis (Bartholdy et al. 2019; Pascoe 2022; Petersson 2023; Somerville et al. 2008).

These findings align with previous meta‐research demonstrating that UC is frequently heterogeneous, poorly defined, and inconsistently reported across clinical contexts (Arienti et al. 2022; Paci et al. 2022). In musculoskeletal and rehabilitation research, variability in comparator interventions has been shown to contribute to heterogeneity in effect estimates and influence conclusions regarding intervention effectiveness (Marriott 2024; Pedersen et al. 2023).

The conclusions of this study were primarily supported by analyses of continuous TIDieR scores. Although categorical classifications may facilitate interpretation, they are inherently less sensitive and may obscure meaningful variability in reporting completeness. In contrast, continuous measures provide a more precise and informative assessment and should be prioritised in future meta‐research. Consistent with this, the dichotomised analysis failed to detect statistically significant differences between groups, whereas the continuous analysis revealed clear differences, suggesting that dichotomisation may underestimate between‐group effects.

The weak and non‐significant association between TIDieR and PEDro scores further reinforces that reporting quality and methodological quality represent distinct dimensions of research rigour. Adequate internal validity does not ensure transparent reporting, highlighting the need to consider reporting completeness as an independent methodological domain (Ioannidis 2005).

No improvement in reporting completeness was observed following the publication of the TIDieR checklist. This suggests that the mere availability of reporting guidelines alone is insufficient to change research practices (Glasziou et al. 2014; L. Turner et al. 2012). Greater efforts are needed to promote adherence, including stricter editorial policies and peer‐review processes. Although we used a pre‐ and post‐2014 approach, this dichotomisation may not capture gradual improvements over time.

From a clinical and research perspective, these findings highlight the need for greater transparency in the description of comparator interventions. Researchers should adopt reporting frameworks such as TIDieR from the study design stage, while journals and reviewers should enforce adherence to reporting standards (Moher et al. 2010).

Only 31.3% of the studies provided an explicit operational definition of UC, with most relying on vague or generic descriptions. This lack of clarity has implications for the design, conduct, and reporting of future trials. The term ‘usual care’ may be inherently ambiguous and insufficient to capture highly heterogeneous interventions. Therefore, comparison groups should be clearly described using standardised frameworks to improve transparency, reproducibility, and overall evidence quality.

Trial registration was inconsistently reported, precluding assessment of its influence on reporting completeness. Key aspects of UC were also frequently underreported, including components, dosage, delivery format, and provider characteristics. This limits replication, assessment of treatment fidelity, and interpretation of whether observed effects are due to the intervention or variability in comparator conditions.

This study has some limitations. We did not examine the characteristics of UC interventions (e.g., type, intensity, or components) or contextual factors (e.g., healthcare setting or country), which may influence reporting completeness. We also did not assess the relationship between reporting completeness of UC and treatment effect estimates or clinical outcomes. Evidence suggests that comparator characteristics can influence effect sizes (Marriott 2024), and poor reporting may obscure between‐group differences. The use of TIDieR for heterogeneous and sometimes unstructured comparator groups may also reduce sensitivity. As this was a study‐level analysis, more granular insights were not possible and ecological bias may be present. Finally, studies using comparator interventions equivalent to usual care but not explicitly as such in titles, abstracts, or indexing fields may not have been retrieved. This reflects the lack of standardised terminology surrounding usual care across clinical trials. Nevertheless, as our objective was to evaluate comparator groups explicitly described as usual care, the inclusion of a comparator‐specific search block was appropriate and aligned with the review question.

Future research should explore the impact of comparator group reporting on treatment effect estimates and the conclusions of systematic reviews. The development or adaptation of reporting guidelines specifically tailored to comparator interventions may further improve transparency in clinical trials.

## 5 Conclusion

Reporting completeness of UC comparator groups in exercise‐based RCTs for knee osteoarthritis is consistently poor, highly heterogeneous and has not improved over time. Although exercise interventions are relatively better described, overall reporting quality remains suboptimal. Continuous analytical approaches appear more sensitive in detecting differences in reporting completeness than dichotomised methods. Improved adherence to reporting frameworks, such as TIDieR, is needed to enhance the interpretability, reproducibility, and validity of evidence synthesis.

## Author Contributions

**José Ribeiro da Silva Neto:** conceptualization, methodology, data curation, formal analysis, investigation, writing – original draft, writing – review and editing. **Lucas Henrique Caldas:** data curation, investigation, screening of studies, writing. **Paula Gabrielly Oliveira Demes:** data curation, investigation, screening of studies, writing. **Natalia Camargo Rodrigues Iosimuta:** supervision, methodology, writing – review and editing. **Ana Carolina Pereira Nunes Pinto:** methodology, writing – review and editing. **Areolino Pena Matos:** conceptualization, methodology, supervision, formal analysis, validation, project administration, writing – original draft, writing – review and editing. All authors contributed to the interpretation of the findings, critically revised the manuscript for important intellectual content, approved the final version of the manuscript, and agreed to be accountable for all aspects of the work.

## Funding

The authors have nothing to report.

## Conflicts of Interest

The authors declare no conflicts of interest.

## Supporting information


Supporting Information S1

**Table S1:** TIDieR scores of both groups and UC description in included RCTs (*n* = 67).

## Data Availability

The data that support the findings of this study are available from the corresponding author upon reasonable request.
