# Supplementary material for: Reporting Completeness of Usual Care Comparator Groups in Exercise‐Based Trials for Knee Osteoarthritis: A Meta‐Research Systematic Review
Source: Musculoskeletal Care. 2026 Jun 28;24(3):e70248. doi: 10.1002/msc.70248 (PMC13310966; doi:10.1002/msc.70248)
Supplement: Supplementary file 1 — Supporting Information S1 [file MSC-24-e70248-s001.docx]

**Supplementary Material**

Because the objective of this review was to evaluate comparator groups explicitly described as usual care or related terminology, the search strategy included a comparator-specific block. Consequently, studies in which comparator interventions functioned as usual care but were not explicitly described using these terms in titles, abstracts, or indexing fields may not have been retrieved. This limitation reflects the lack of standardization and conceptual ambiguity surrounding usual care interventions across clinical trials.

**Electronic Search Strategies**

| **Database** | | **Platform** | **Date** | **Records retrieved** |
| --- | --- | --- | --- | --- |
| MEDLINE | PubMed | | 31 December 2024 | 226 |
| EMBASE | Elsevier | | 31 December 2024 | 378 |
| CENTRAL | Cochrane Library | | 31 December 2024 | 577 |
| CINAHL | EBSCO | | 31 December 2024 | 119 |
| SPORTDiscus | EBSCO | | 31 December 2024 | 219 |
| PEDro | PEDro | | 31 December 2024 | 712 |
| **Total** |  | |  | **2231** |

### **Note on search strategy**

The objective of this review was to identify randomized controlled trials of exercise-based interventions for knee osteoarthritis that included a comparator group explicitly described as usual care or related terminology (e.g., standard care, routine care, treatment as usual). Therefore, the search strategy incorporated a comparator-specific block, resulting in a narrower retrieval than conventional searches designed to identify all exercise-based trials in knee osteoarthritis.

# MEDLINE (PubMed)

**Date searched:** 31 December 2024

**Records retrieved:** 226

#1 "Osteoarthritis, Knee"[Mesh]

OR (Knee Osteoarthritides)

OR (Knee Osteoarthritis)

OR (Osteoarthritis of Knee)

OR (Osteoarthritis of the Knee)

#2 "Exercise"[Mesh]

OR Exercise (Physical Activity)

OR (Activities, Physical)

OR (Activity, Physical)

OR (Physical Activities)

OR (Exercise, Physical)

OR (Exercises, Physical)

OR (Physical Exercise)

OR (Physical Exercises)

OR (Acute Exercise)

OR (Acute Exercises)

OR (Exercise, Acute)

OR (Exercises, Acute)

OR (Exercise, Isometric)

OR (Exercises, Isometric)

OR (Isometric Exercises)

OR (Isometric Exercise)

OR (Exercise, Aerobic)

OR (Aerobic Exercise)

OR (Aerobic Exercises)

OR (Exercises, Aerobic)

OR (Exercise Training)

OR (Exercise Trainings)

OR (Training, Exercise)

OR (Trainings, Exercise)

OR "Exercise Therapy"[Mesh]

OR (Remedial Exercise)

OR (Exercise, Remedial)

OR (Exercises, Remedial)

OR (Remedial Exercises)

OR (Therapy, Exercise)

OR (Exercise Therapies)

OR (Therapies, Exercise)

OR (Rehabilitation Exercise)

OR (Exercise, Rehabilitation)

OR (Exercises, Rehabilitation)

OR (Rehabilitation Exercises)

#3 "Standard of Care"[Mesh]

OR (Usual care)

OR (Care Standard)

OR (Care Standards)

OR (Standards of Care)

OR (Usual standard care)

OR (Standard conservative therapy)

OR (Care-as-usual)

OR (Nonstandardized care)

OR (Routine treatment)

OR (Treatment-as-usual)

#4 ((clinical[Title/Abstract] AND trial[Title/Abstract])

OR clinical trials as topic[MeSH Terms]

OR clinical trial[Publication Type]

OR random*[Title/Abstract]

OR random allocation[MeSH Terms]

OR therapeutic use[MeSH Subheading])

#5 #1 AND #2 AND #3 AND #4

**EMBASE**

**Date searched:** 31 December 2024

**Records retrieved:** 378

The search strategy was adapted to the EMTREE vocabulary and database-specific syntax while preserving the same concepts:

- Population: knee osteoarthritis
- Intervention: exercise/exercise therapy
- Comparator: usual care and related terminology
- Study design: randomized and clinical trials

Final search:

#1 AND #2 AND #3 AND #4

**CENTRAL**

**Date searched:** 31 December 2024

**Records retrieved:** 577

The search strategy was adapted to CENTRAL indexing and syntax while preserving the same concepts

Final search:

Osteoarthritis, Knee* OR Knee Osteoarthritides OR Knee Osteoarthritis OR Osteoarthritis of Knee OR Osteoarthritis of the Knee

AND

Exercise* OR Physical Activity OR Activities, Physical OR Activity, Physical OR Physical Activities OR (Exercise, Physical) OR (Exercises, Physical) OR (Exercises, Physical) OR (Physical Exercise) OR (Physical Exercises) OR (Acute Exercise) OR (Acute Exercises) OR (Exercise, Acute) OR (Exercises, Acute) OR (Exercise, Isometric) OR (Exercises, Isometric) OR (Isometric Exercises) OR (Isometric Exercise) OR (Exercise, Aerobic) OR (Aerobic Exercise) OR (Aerobic Exercises) OR (Exercises, Aerobic) OR (Exercise Training) OR (Exercise Trainings) OR (Training, Exercise) OR (Trainings, Exercise) OR "Exercise Therapy" OR (Remedial Exercise) OR (Exercise, Remedial) OR (Exercises, Remedial) OR (Remedial Exercises) OR (Therapy, Exercise) OR (Exercise Therapies) OR (Therapies, Exercise) OR (Rehabilitation Exercise) OR (Exercise, Rehabilitation) OR (Exercises, Rehabilitation) OR (Rehabilitation Exercises)

AND

Standard of Care OR Usual Care OR Care Standard OR Care Standards OR Standards of Care OR Usual standard care Standard care

AND

Clinical Trials

# CINAHL

**Date searched:** 31 December 2024

**Records retrieved:** 119

The search strategy was adapted to CINAHL subject headings and database-specific syntax while preserving the same concepts

Final search:

Osteoarthritis, Knee* OR Knee Osteoarthritides OR Knee Osteoarthritis OR Osteoarthritis of Knee OR Osteoarthritis of the Knee AND Exercise* OR Physical Activity OR Activities, Physical OR Activity, Physical OR Physical Activities OR (Exercise, Physical) OR (Exercises, Physical) OR (Exercises, Physical) OR (Physical Exercise) OR (Physical Exercises) OR (Acute Exercise) OR (Acute Exercises) OR (Exercise, Acute) OR (Exercises, Acute) OR (Exercise, Isometric) OR (Exercises, Isometric) OR (Isometric Exercises) OR (Isometric Exercise) OR (Exercise, Aerobic) OR (Aerobic Exercise) OR (Aerobic Exercises) OR (Exercises, Aerobic) OR (Exercise Training) OR (Exercise Trainings) OR (Training, Exercise) OR (Trainings, Exercise) OR "Exercise Therapy" OR (Remedial Exercise) OR (Exercise, Remedial) OR (Exercises, Remedial) OR (Remedial Exercises) OR (Therapy, Exercise) OR (Exercise Therapies) OR (Therapies, Exercise) OR (Rehabilitation Exercise) OR (Exercise, Rehabilitation) OR (Exercises, Rehabilitation) OR (Rehabilitation Exercises) AND Standard of Care OR Usual Care OR Care Standard OR Care Standards OR Standards of Care OR Usual standard care AND Clinical trial OR clinical trials OR random* OR random allocation OR therapeutic use

# SPORTDiscus

**Date searched:** 31 December 2024

**Records retrieved:** 219

The search strategy was adapted to SPORTDiscus indexing and syntax while preserving the same concepts

Final search:

"Osteoarthritis, Knee"[Mesh] OR (Knee Osteoarthritides) OR (Knee Osteoarthritis) OR (Osteoarthritis of Knee)

AND

Exercise* OR Physical Activity OR Activities, Physical OR Activity, Physical OR Physical Activities OR (Exercise, Physical) OR (Exercises, Physical) OR (Exercises, Physical) OR (Physical Exercise) OR (Physical Exercises) OR (Acute Exercise) OR (Acute Exercises) OR (Exercise, Acute) OR (Exercises, Acute) OR (Exercise, Isometric) OR (Exercises, Isometric) OR (Isometric Exercises) OR (Isometric Exercise) OR (Exercise, Aerobic) OR (Aerobic Exercise) OR (Aerobic Exercises) OR (Exercises, Aerobic) OR (Exercise Training) OR (Exercise Trainings) OR (Training, Exercise) OR (Trainings, Exercise) OR "Exercise Therapy" OR (Remedial Exercise) OR (Exercise, Remedial) OR (Exercises, Remedial) OR (Remedial Exercises) OR (Therapy, Exercise) OR (Exercise Therapies) OR (Therapies, Exercise) OR (Rehabilitation Exercise) OR (Exercise, Rehabilitation) OR (Exercises, Rehabilitation) OR (Rehabilitation Exercises)

AND

("usual care" OR "standard care" OR "standard of care" OR "routine care" OR "conventional care")

AND

(random* OR randomized OR randomised OR "clinical trial*" OR "controlled trial*" OR trial OR trials)

**PEDro**

**Date searched:** 31 December 2024

**Records retrieved:** 712

Search terms:

knee osteoarthritis

usual care

exercise

Filters:

Clinical trial
