# Supplementary material for: Reporting Completeness of Usual Care Comparator Groups in Exercise‐Based Trials for Knee Osteoarthritis: A Meta‐Research Systematic Review
Source: Musculoskeletal Care. 2026 Jun 28;24(3):e70248. doi: 10.1002/msc.70248 (PMC13310966; doi:10.1002/msc.70248)
Supplement: Supplementary file 2 — Table S1: TIDieR scores of both groups and UC description in included RCTs (n = 67). [file MSC-24-e70248-s002.docx]

(Supplementary material)

| **Supplementary Table 1 - TIDieR scores of both groups and UC description in included RCTs (n = 67).** | | | | | | |
| --- | --- | --- | --- | --- | --- | --- |
|  | **Exercise group** | |  | **Usual care group** | | |
| **Author(s)** | **Score** | **Classification** |  | **Score** | **Classification** | **Description** |
| (Villadsen, Overgaard, Holsgaard-Larsen, Christensen, & Roos, 2014) | 7 | HQ |  | 3 | LQ | Education |
| (Skou et al., 2015) | 6 | HQ |  | 4 | LQ | Informational leaflet |
| (Pisters, Veenhof, Schellevis, De Bakker, & Dekker, 2010) | 6 | HQ |  | 3 | LQ | Information and advice |
| (Allen et al., 2016) | 2 | LQ |  | 1 | LQ | Standard treatments in primary care (undefined) |
| (Kim & Kim, 2020) | 5 | LQ |  | 1 | LQ | Undefined |
| (Abbott et al., 2013) | 3 | LQ |  | 1 | LQ | Routine care by family physicians and other healthcare providers |
| (Wallis et al., 2017) | 5 | LQ |  | 2 | LQ | Non-operative management for pain and symptom control including pharmacological and non-pharmacological treatment |
| (Murphy et al., 2016) | 5 | LQ |  | 2 | LQ | Maintain UC (undefined) and telephone call follow-up |
| (Williamson, Wyatt, Yein, & Melton, 2007) | 4 | LQ |  | 2 | LQ | Leaflets and advice |
| (Knoop et al., 2020) | 7 | HQ |  | 2 | LQ | Exercises |
| (Runhaar et al., 2019) | 1 | LQ |  | 1 | LQ | Healthy lifestyle |
| (Saw, Parker, & Edries, 2015) | 6 | HQ |  | 1 | LQ | Medical care |
| (Hurley et al., 2007) | 5 | LQ |  | 1 | LQ | Medical intervention in primary care |
| (Bruce-Brand et al., 2012) | 5 | LQ |  | 1 | LQ | Education, weight loss, pharmacological treatment, and physical therapy |
| (Ravaud, 2004) | 5 | LQ |  | 1 | LQ | Medical care |
| (Isaramalai et al., 2018) | 6 | HQ |  | 1 | LQ | UC (undefined) and education |
| (Gohir, Eek, Kelly, Abhishek, & Valdes, 2021) | 4 | LQ |  | 1 | LQ | Exercises and information |
| (Nigam, Satpute, & Hall, 2021) | 5 | LQ |  | 5 | LQ | Exercise and moist heat |
| (Kloek et al., 2018) | 5 | LQ |  | 4 | LQ | Physical therapy |
| (Knoop et al., 2022) | 3 | LQ |  | 2 | LQ | Exercises |
| (Marra et al., 2012) | 1 | LQ |  | 2 | LQ | Informational leaflets |
| (Segal et al., 2015) | 3 | LQ |  | 1 | LQ | OA self-management |
| (Cochrane, Davey, & Matthes Edwards, 2005) | 6 | HQ |  | 1 | LQ | Telephone call follow-up |
| (Heuts et al., 2005) | 1 | LQ |  | 2 | LQ | OA self-management |
| (Karadağ, Taşci, Doğan, Demir, & Kiliç, 2019) | 1 | LQ |  | 1 | LQ | Medical recommendations |
| (Kovar et al., 1992) | 3 | LQ |  | 1 | LQ | Standard routine medical care |
| (Saw, Kruger-Jakins, Edries, & Parker, 2016) | 6 | HQ |  | 1 | LQ | Medical recommendations |
| (Munukka et al., 2016) | 4 | LQ |  | 1 | LQ | Maintain habitual physical activity level |
| (Hunter et al., 2023) | 4 | LQ |  | 1 | LQ | Medical appointments |
| (Mete & Sari, 2022) | 2 | LQ |  | 3 | LQ | Conventional physical therapy (electrotherapy and exercise) |
| (Singh, Pattnaik, Mohanty, & Ganesh, 2016) | 4 | LQ |  | 2 | LQ | Exercises |
| (Multanen et al., 2014) | 3 | LQ |  | 1 | LQ | Undefined |
| (Rewald et al., 2020) | 6 | HQ |  | 2 | LQ | Exercise therapy or complementary therapy |
| (Veenhof et al., 2007) | 3 | LQ |  | 3 | LQ | Guidelines advice |
| (Skou ST; Roos EM; Simonsen O; Laursen MB; Rathleff MS; Arendt-Nielsen L; Rasmussen S, 2016) | 4 | LQ |  | 2 | LQ | Informational leaflets |
| (Dziedzic et al., 2018) | 3 | LQ |  | 1 | LQ | Undefined |
| (Allen et al., 2017) | 1 | LQ |  | 1 | LQ | Guidelines advice |
| (Skou ST; Rasmussen S; Laursen MB; Rathleff MS; Arendt-Nielsen L; Simonsen O; Roos EM, 2015) | 4 | LQ |  | 2 | LQ | Informational leaflets |
| (An, Ryu, Lyu, Yi, & Lee, 2021) | 6 | HQ |  | 1 | LQ | Health guidance and exercises |
| (C. Xiao, Zhuang, & Kang, 2020) | 4 | LQ |  | 5 | LQ | Conventional physical therapy |
| (Quilty, Tucker, Campbell, & Dieppe, 2003) | 3 | LQ |  | 1 | LQ | Non-standard physical therapy treatment |
| (Lin, Lin, Lin, & Jan, 2009) | 4 | LQ |  | 1 | LQ | No intervention |
| (Ye et al., 2020) | 4 | LQ |  | 1 | LQ | Maintain normal lifestyle |
| (Wang, Belza, Elaine Thompson, Whitney, & Bennett, 2007) | 4 | LQ |  | 1 | LQ | Asked to continue physical activities |
| (Henriksen et al., 2014) | 6 | HQ |  | 1 | LQ | No attention from the study (no intervention) |
| (Thomas et al., 2002) | 4 | LQ |  | 1 | LQ | No intervention |
| (Jan, Lin, Liau, Lin, & Lin, 2008) | 7 | HQ |  | 1 | LQ | No intervention |
| (Veenhof et al., 2006) | 4 | LQ |  | 1 | LQ | Recommendations and advice on exercises |
| (Simão et al., 2012) | 4 | LQ |  | 1 | LQ | Maintain lifestyle |
| (Choi, Kim, Hwang, Moon, & Choi, 2015) | 3 | LQ |  | 2 | LQ | Conventional physical therapy (ultrasound and hot pack) |
| (Rafiq MT; Hamid MSA; Hafiz E, 2021) | 2 | LQ |  | 2 | LQ | Daily care as UC (undefined) |
| (Onwunzo, Igwe, Umunnah, Uchenwoke, & Ezugwu, 2021) | 3 | LQ |  | 2 | LQ | Daily care instructions (undefined) |
| (Swank et al., 2011) | 5 | LQ |  | 3 | LQ | Undefined |
| (Mazloum, Rabiei, Rahnama, & Sabzehparvar, 2018) | 4 | LQ |  | 1 | LQ | Maintain daily routines and prohibited from participating in any exercises or sports activities |
| (Z. Xiao & Li, 2021) | 1 | LQ |  | 1 | LQ | No physical exercise |
| (Chang, Liou, Chen, Huang, & Chang, 2012) | 3 | LQ |  | 2 | LQ | Conventional modality treatments (undefined) |
| (Øiestad et al., 2023) | 6 | HQ |  | 1 | LQ | No intervention |
| (Ahmad et al., 2023) | 1 | LQ |  | 3 | LQ | Usual physical therapy treatment (hot packs, cryotherapy, ultrasound, and electrical stimulation) |
| (Alghadir AH; Anwer S; Sarkar B; Paul AK; Anwar D, 2019) | 4 | LQ |  | 1 | LQ | Undefined |
| (Dantas et al., 2023) | 6 | HQ |  | 1 | LQ | Pharmacological treatment and/or physical activity |
| (Rêgo et al., 2023) | 5 | LQ |  | 1 | LQ | No physical intervention or therapeutic guidance |
| (Knoop et al., 2023) | 3 | LQ |  | 1 | LQ | Physical therapy |
| (Sadeghi et al., 2023) | 1 | LQ |  | 1 | LQ | No intervention |
| (Terradas-Monllor et al., 2023) | 6 | HQ |  | 2 | LQ | Education and physical therapy |
| (Walrabenstein et al., 2023) | 1 | LQ |  | 1 | LQ | Undefined |
| (Østerås et al., 2024) | 1 | LQ |  | 1 | LQ | Family physician visits, pharmacological therapy |
| (Foster, Nicholls, Holden, Healey, & Hay, 2023) | 2 | LQ |  | 4 | LQ | Advice and exercise |

**HQ: High Quality; LQ: Low Quality.**
